# Age-dependent changes in the power spectrum conflate composite scores to assess brain frailty

**DOI:** 10.1016/j.cnp.2025.06.002

**Published:** 2025-06-19

**Authors:** Julian Ostertag, Aleksandra Migal, David P. Obert, Gerhard Schneider, Pablo Sepúlveda, Matthias Kreuzer

**Affiliations:** a Department of Anesthesiology and Intensive Care, School of Medicine and Health, Technical University of Munich, Ismaninger Str. 22, Munich 81675, Germany; b Department of Anesthesiology, Hospital Base San José, Universidad Austral, Av. Guillermo Bühler 1765, Osorno, Chile

**Keywords:** Anesthesia, EEG, TCI, Age, Spectral power

## Abstract

**Objective:**

Evaluating age-related dependencies in the electroencephalogram (EEG) during induction of general anesthesia and their impact on composite scores used to assess frailty.

**Methods:**

A composite score was derived from spectral edge frequency, total power, alpha power, and the effect-site concentration (Ce) of propofol. All these parameters are influenced by age, brain health, and dosage and speed of drug administration. Correlation coefficients and variance inflation factors were used to determine multicollinearity. Differences in the spectral EEG features of patients with “high” and “low” composite scores were assessed by the area under the receiver operator characteristic curve (AUC) as the statistical test.

**Results:**

The EEG features, total power and alpha power, were strongly correlated (*ρ* = 0.82). But alpha power (*ρ* = 0.17) and total power (*ρ* = 0.2) were only weakly correlated with propofol, indicating a weak model. Additionally, the composite score showed a moderate negative correlation with age (*ρ* = -0.44). We also observed significant and strong (AUC *<* 0.3) differences in total power and the power of all EEG bands except gamma between patients with a ”high” and a ”low” score before loss of responsiveness (LOR).

**Conclusion:**

Patient age significantly influences EEG-based parameters within the score. Importantly, significant differences in spectral EEG features between the groups were already observable before LOR. These differences could allow for early assessment of a patient’s brain state and to titrate anesthetic dose before LOR. The study also shows that age should be considered as it can drive models for ”frailty”.

**Significance:**

Age moderately influenced all subcomponents and should consequently be factored into score interpretation.

## Highlights

• Age can conflate composite scores containing EEG-related parameters.
• Patients with high composite scores showed differences in EEG-related parameters compared to those with low scores.
• Differences are detectable already before loss of responsiveness that could allow adjustments of anesthesia navigation.

## 1 Introduction

Monitoring the brain using the electroencephalogram (EEG) can provide the anesthesiologist with valuable information regarding the level and quality of anesthesia. Similarly, pharmacokinetic/pharmacodynamic (PKPD) modeling in combination with or without target-controlled infusions (TCI) offers another means of monitoring anesthesia. Yet both approaches also have limitations. When discussing ”EEG-guided” anesthesia, the main focus is on processed EEG indices as presented by commercial monitoring systems (Hao et al., 2023). The overwhelming majority of users focuses on this information while neglecting other sources like the raw EEG and the spectral representation of the EEG (Aasheim et al., 2024). This focus on a processed number can influence the anesthesia navigation as the companies provide an ”adequate anesthesia” index range for their monitor, e.g. 60 to 40 for the bispectral index (BIS) monitor (Avidan et al., 2008). Further studies describe index values of ”light anesthesia” to be favored over index values of ”deep anesthesia” (Sumner et al., 2023). PKPD models, on the other hand, may not sufficiently describe the nonlinear behavior of brain dynamics (Sepúlveda et al., 2023). The models may overpredict the effect, which could potentially lead to misreferences and cause overdosing. But over the last years, spectral EEG information in the form of graphical representations as density spectral arrays or EEG band-powers have been increasingly used to estimate the brain state under anesthesia (Purdon et al., 2015a, Purdon et al., 2015b). For GABAergic anesthetics like propofol or fluranes, the oscillatory activity in the delta- and alpha-band of the EEG became of high interest as strong oscillations indicate an idling thalamocortical communication that may reflect an adequate anesthesia level (Franks, 2008). Although these EEG features are desirable, their appearance can change with age. The EEG of older patients is generally of lower amplitude, resulting in lower power in individual frequency bands and consequently lower overall power (Purdon et al., 2015a, Purdon et al., 2015b, Kreuzer et al., 2020, Schultz et al., 2004, Schnider et al., 1999). In relative terms, the intraoperative EEG of geriatric patients looks somewhat more awake (Kreuzer et al., 2020) as it is faster and of lower amplitude. This change in EEG can lead to higher processed indices as the monitor interprets the faster and lower amplitude EEG as “more awake” (Ni et al., 2019, Obert et al., 2021). Hence, older patients may be at a higher risk of being overdosed when the anesthesiologist tries to target a certain index. Further, the indices seem insensitive to changes in alpha oscillatory activity (Kinateder et al., 2022) as they disregard this range and focus on the change of faster frequencies or the ratio in power between slower (delta) and faster (beta, gamma) frequencies. But alpha oscillatory activity is a distinct feature of adequate anesthesia (Purdon et al., 2015a, Purdon et al., 2015b) and may also carry information about a possible frail brain. Lower intraoperative alpha-band power (Gutierrez et al., 2019) as well as low or absent alpha-band power during anesthesia emergence (Hesse et al., 2019, Lutz et al., 2022) seems to be associated with a higher delirium risk as well as with lower preoperative cognitive scores (Giattino et al., 2017). Hence, the EEG may carry information about the brain’s cognitive state or its ”frailty”. As of now, there is no consensus about a definition of frailty (Morley et al., 2013), however in the anesthesia context, this frailty is often indirectly assessed by means of sensitivity to propofol, the susceptibility to EEG burst suppression, or the occurrence of postoperative neurocognitive disorders such as delirium. Because of these age-induced and frailty-associated intraoperative EEG features, composite scores have been developed to evaluate ”brain frailty” (Touchard et al., 2019). Touchard and colleagues described a composite score including total power, alpha-band power and propofol dosage to assess the fragility of patients undergoing general anesthesia. The authors selected these parameters based on their own reasoning and existing literature; however, the score has not yet been validated. They suggested, that a low propofol concentration to reach a spectral edge frequency of 8–13 Hz, a low total EEG power, and a low alpha-band power may indicate a fragile brain. Additionally, they found that patients with a low score, indicative of a ”frailer” brain, had a higher incidence of burst suppression during anesthesia induction. Research suggests that burst suppression is a marker of a frail brain, which is more susceptible to postoperative neurocognitive disorders (Soehle et al., 2015, Fritz et al., 2016). In this analysis, we want to critically evaluate the use of composite scores and their sub-components in anesthesia monitoring. Unlike previous studies, staged on loading boluses or high TCI effect site concentrations, we propose a slow, progressive, fine titration scenario. This may avoid the biases of propofol bolus overdose and the overprediction of the PKPD models observed after bolus induction, ultimately impacting different mechanisms of inducing unresponsiveness (Sepulveda et al., 2020). Studies with slow inductions show not only more intense alpha-band power, but normalized to full power, it is observed that elderly patients would have an alpha-band similar to young patients (Obert et al., 2024). Adding age as a confounding factor is another crucial element of this study. Furthermore, we used the composite score to separate patients into a high- and low-scoring group. This allows us to evaluate which other parameters differ between the groups and when these differences arise during the anesthetic procedure. Similarly, grouping could allow for adjustments in anesthesia titration by identifying differences in brain activity indicative of varying anesthetic needs. While being reproducible during slow, progressive induction, our composite score did not correlate with burst suppression, a proposed marker of brain fragility. Instead, the composite score was driven by age, thus limiting its specificity and usefulness for individualized anesthesia care or frailty estimations.

## 2 Methods

### 2.1 Study protocol

Our retrospective analysis is based on EEG recordings collected during several studies conducted at the Clínica Alemana Santiago and Hospital Base San José Osorno in Chile. The local ethics committee approved all studies, and all patients gave informed consent. The studies were registered at Clinical Trials under NCT05425069(Mehler et al., 2024), NCT03140982(Obert et al., 2020). Patients between 18 and 91 years of age scheduled for elective surgery were included. Patients did not have any decompensated systemic pathology, no neurocognitive compromise, and no history of drug abuse or chronic use of benzodiazepines. Neurosurgical or emergency surgery, as well as ASA III-IV patients, were excluded. The patients were not premedicated. An adequate venous line, standard hemodynamic monitoring, and frontal EEG monitor were installed in the operating room as recommended by the guidelines. After a dose of 40 mg lidocaine to avoid the pain of the propofol injection, we proceeded to infuse propofol with a flow rate of 10 mg/kg/h in those under 65 years of age and 8 mg/kg/h in those over 65 years of age, until loss of responsiveness (LOR) defined as loss of response to loud call and tap on the shoulder. One minute after LOR, we switched to (effect site) TCI mode and kept the effect site concentration as calculated by the Schnider model constant. Then remifentanil infusion was started with an effect site concentration of 4.5 ng/ml following Minto modelling and after 90 seconds rocuronium 0.6 mg/kg was injected. After waiting for the appropriate latency time, tracheal intubation was performed. Continuous bilateral EEG was recorded throughout this process and throughout the surgery.

### 2.2 EEG recording and analysis

For our analyses, we used raw EEG data from 75 patients undergoing general anesthesia (Mehler et al., 2024, Obert et al., 2020). EEG was recorded using SEDLine (N = 63) or BIS monitors with sample rates of 178.15 Hz or 128 Hz. The raw EEG data was filtered using a 0.5 Hz high-pass and a 40 Hz low-pass zero phase shift filter. Data acquired with SEDLine monitors was re-sampled to 128 Hz to match the sample rate of the BIS recordings. From each recording, 5 min before until 20 min after loss of responsiveness were extracted. We calculated the power spectral density (PSD) from single channels using MATLAB’s *pwelch* function for 10 s epochs, with NFFT of 256, resulting in a frequency resolution of 0.5 Hz. From the PSD, we extracted the band power in the delta-band (0.5–4 Hz), theta-band (4–8 Hz), alpha-band (8–13 Hz), beta-band (13–30 Hz), and gamma-band (30–40 Hz) as well as the spectral edge frequency (SEF95). We also normalized power by correcting for the overall power present at every given time point to evaluate the EEG architecture, i.e., the contribution of each frequency’s power to the total power. In our analysis, the SEF95 defines the frequency below which 95 % of the total power in the EEG spectrum from 0.5 Hz to 40 Hz is concentrated and is a well-established measure of anesthetic effects (Schwender et al., 1996). But as with all indices, the SEF dismisses a lot of information, and the shape of the power spectrum can not be reproduced from the SEF alone.

### 2.3 Calculation of the composite score

The composite score mentioned in the introduction, BPTIVA, consists of three parameters: total power, alpha-band power, and propofol effect-site concentration (Touchard et al., 2019). The propofol concentration was ranked on a scale from 1 to 3, the total power from 1 to 4, and the alpha power from 1 to 5, leading to a minimum score of 3 and a maximum score of 12 (**Table S1**). A rising score corresponds to increasing sub-parameter values. As per the original publication by Touchard and colleagues (Touchard et al., 2019), we sought to identify a specific period after loss of responsiveness where the SEF95 ranged between 8–13 Hz for at least 5 min. However, these prerequisites were only met by 3 out of 75 patients. Two modifications had to be applied to maximize the number of patients for whom BPTIVA scores could be calculated. Firstly, we extended the windows of allowed SEF95s to 8–20 Hz. Secondly, we allowed 20 percent of SEF95s to be outside the window, effectively extending the search window to 6 min, from which 5 min of valid data points were extracted. With these modifications, we could calculate a score for 70 out of the 75 patients.

### 2.4 Statistical analysis

Because we conducted a retrospective study, we present p-values and effect sizes as recommended (Sullivan and Feinn, 2012). We did not perform a post hoc power analysis, as it is considered redundant and potentially misleading when confidence intervals and effect sizes are reported (Hoenig, 2001). Additionally, due to the retrospective, exploratory nature, we do not expect small changes to be detectable, which however, does not exclude them from being present. Based on the mean value of the composite score, patients were grouped into ”high” and ”low” scoring patients.

Differences in demographic variables (age, weight, height, ASA status, propofol concentration at loss of responsiveness, and SEF95) were assessed using the area under the receiver operator characteristic curve (AUC) as effect size and p-values from a Wilcoxon’s Rank-Sum test. We present median values as well as 25th and 75th percentiles to show the distribution within the respective groups. AUC values can be interpreted as the distance from 0.5. Higher distances mean better separation between the groups. AUC values were bootstrapped 5000 times to find the boundaries of a 95 % confidence interval. If the confidence interval does not contain AUC = 0.5, differences can be considered statistically significant (Hentschke and Stüttgen, 2011). AUC values are symmetrical around 0.5. As a rule of thumb, an *AUC >* 0*.*7 or *AUC <* 0*.*3 as effect size can both be considered ”acceptable” (Mandrekar, 2010).

We calculated correlation coefficients and the respective 95 % confidence intervals using MATLAB’s *corrcoef* function to see how age influences individual subcomponents of the composite score. All sub-components, total power, alpha power (Purdon et al., 2015a, Purdon et al., 2015b), and propofol concentration at LOC (Yang et al., 2022), and the original BPTIVA score (Touchard et al., 2019) have been shown to correlate with age. Correlation coefficients were interpreted using conventional cutoff values as provided by Schober and colleagues (Schober et al., 2018). Following this convention, correlation coefficients between 0 and 0.1 are considered negligible, 0.1–0.39 are weak, 0.4–0.69 are moderate, 0.7–0.89 are strong, and 0.9–1 are very strong. Additionally, we supply the p-value from the hypothesis test that there is ”no correlation”. Further, we calculated the variance inflation factor to capture the amount of multicollinearity in a set of regression variables. To interpret the variance inflation factor, we used the conventional cutoff values of 1 = no correlation, 1–5 = moderate correlation, and above 5 = high correlation.

AUC values were again used to assess the amount of separation between the groups for EEG-derived parameters 3 min before loss of responsiveness and overall epochs.

To reduce the risk of discussing false positive results, we only considered a result significant if at least two neighboring time points indicated relevant differences. To analyse non-dichotomized data, we used the prediction probability (PK) as a generalization of the area under the receiver operating characteristic curve that fits the polytomous composite score (Smith et al., 1996). For AUC and PK values, the same interpretation is applied. Additionally, in our context, values above 0.5 meant that higher scores are associated with higher power, while values below 0.5 meant that higher scores are associated with lower power.

## 3 Results

### 3.1 Demographics

With our approach, we could classify our patients with composite scores ranging from 4 to 12 and a mean score of 9.4. With the mean score defined as the cutoff score, 42 patients were categorized as ”high scorers” and 28 patients were ”low scorers”. After dichotomization, we found that ”low scorers” were significantly older, had a significantly higher ASA score, received significantly less propofol, and had a significantly lower SEF95 (see Table 1).

**Table 1:** Demographics and statistical comparison of the high and low scorer groups. AUC values as the effect size are symmetrical around 0.50, therefore both AUC < 0.30 or > 0.70 are considered acceptable. Median and 25th & 75th percentiles (in brackets) are reported.

**Variable**	**High scorers**	**Low scorers**	**Effect Size**	**Effect**	**P-Value**
**Age**	59	73.5	0.772	acceptable	0.001*
	[41;73]	[71;78]	[0.654;0.876]		
**Weight**	70	73	0.543	none	0.552
	[62;82]	[65;79.5]	[0.408;0.674]		
**Height**	162	162.5	0.499	none	0.995
	[155;170]	[157;167.5]	[0.37;0.636]		
**ASA**	2	2	0.711	acceptable	0.002*
	[1;2]	[2;3]	[0.598;0.815]		
**Propofol (LOR)**	2.9	2.35	0.265	acceptable	0.001*
	[2.6;3.2]	[2.05;2.75]	[0.148;0.395]		
**SEF**	16.8	14.5	0.278	acceptable	0.002*
	[15.3;17.3]	[12.95;16.45]	[0.156;0.406]		

### 3.2 Composite score analysis

**Fig. 1:**
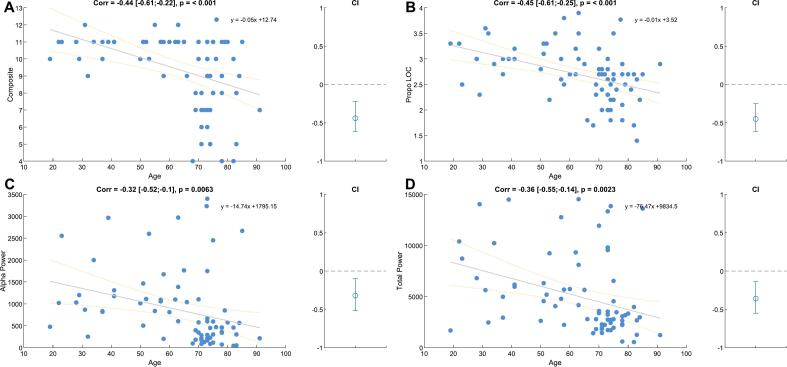
**Relationship between our composite score and its components and age. (A)** The composite score’s correlation coefficient was *ρ* **= -0.44 [-0.61;-0.22]. (B)** The propofol concentrations correlation coefficient was *ρ* **= -0.45 [-0.61; −0.25]. (C)** The alpha-band power’s correlation coefficient was *ρ* **= -0.32 [-0.52; −0.10. (D)** The total power’s correlation coefficient was *ρ* **= -0.36 [-0.55; −0.14]**.

From the sub-components, alpha and total power were strongly positively correlated with each other (*ρ* = 0.82 [0.73; 0.89], *p <* 0*.*001), while alpha power and propofol concentration (*ρ* = 0.17 [-0.07;0.39], *p* = 0*.*160) and total power and propofol concentration (*ρ* = 0.2 [-0.03;0.42], *p* = 0*.*092) were only weakly positively correlated. These relationships result in a variance inflation factor of 1.043 for propofol concentration, 3.071 for alpha power, and 3.110 for total power. We further explored the relationship between our composite score and the age of the patients (Fig. 1A). Our analysis revealed a moderate negative correlation between the composite score and age, as indicated by a Pearson correlation coefficient of ρ =  − 0.44 [-0.61;-0.22] (p < 0.001). The equation of the linear fit was: composite score =  − 0.05 ∗ age + 12.74 (R^2^ = 0.19). Additionally, propofol concentration at LOR (ρ = -0.45 [-0.61; −0.25], p < 0.001), alpha power (ρ = -0.32 [-0.52; −0.10], p < 0.006) and total power (ρ −0.36 [-0.55; −0.14], p < 0.002) also showed a moderate negative correlation with age (Fig. 1B-D), as could be expected from existing literature (Purdon et al., 2015a, Purdon et al., 2015b, Kreuzer et al., 2020, Schultz et al., 2004).

### 3.3 Dichotomized analyses

After the dichotomization into the high (*composite score >*= 9*.*4*,N* = 43) and low scores (*composite score <* 9*.*4*,N* = 27), we derived the AUC of each component and the other band-powers to separate ”high-score” and ”low-score” patients and we assessed temporal evolution of EEG-derived parameters throughout the induction period.

#### 3.3.1 Separation performance within the EEG interval used for score calculation

**Fig. 2:**
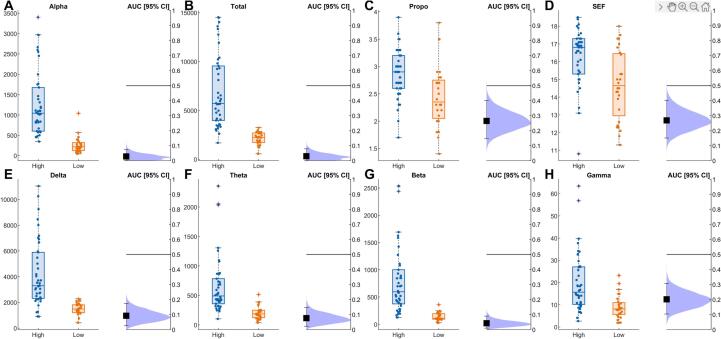
**Boxplots and ROC curves when separating ”high-score” and ”low-score” patients at the time of BPTIVA calculation. AUC values as the effect size are symmetrical around 0.50, therefore both AUC < 0.30 or > 0.70 are considered acceptable**.

For each of the observed features, we found significant differences with *AUC <* 0*.*3, indicative of an “acceptable” effect. For the total EEG power as well as all band powers except gamma-band power the *AUC* was even below 0.1, pointing towards a very strong effect. The corresponding box plots and AUC distributions are presented in Fig. 2.

#### 3.3.2 EEG features during anesthesia induction

**Fig. 3:**
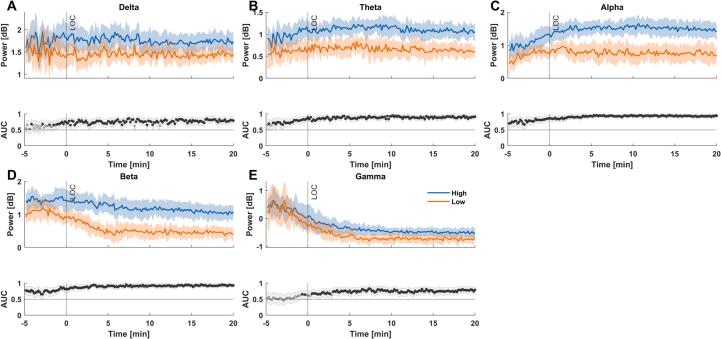
**Differences in absolute EEG band power between ”high-score” and ”low-score” patients during anesthesia induction. (A)** ”High-score” patients had significantly higher delta-band power throughout induction except during the first minutes. **(B)** ”High-score” patients had significantly higher theta-band power throughout induction. **(C)** ”High-score” patients had significantly higher alpha-band power throughout induction. **(D)** “High-score” patients had significantly higher beta-band power throughout induction. **(E)** ”High-score” patients had significantly higher gamma-band power starting around the time of LOR. **(F)** AUC values as the effect size are symmetrical around 0.50, therefore both AUC < 0.30 or > 0.70 are considered acceptable.

The temporal analysis at the group level revealed that already throughout the induction period, ”high-score” patients had significantly higher absolute power as early as 5 min before LOR in theta-, alpha- and beta-band. For delta-bandpower, this pattern was visible around 2–3 min before LOR, and for gamma-bandpower around and after the time of LOR. (Fig. 3). The ”high-score” patients had significantly higher power in most frequency bands throughout induction, with only the gamma-band and delta-band revealing no significant differences during the induction’s early stage.

**Fig. 4:**
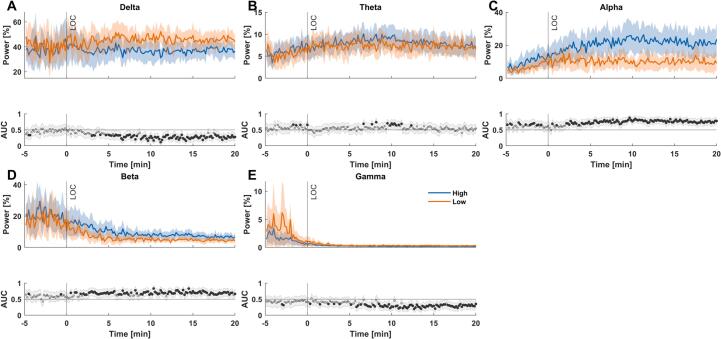
**Differences in relative EEG band power between ”high-score” and ”low-score” patients during anesthesia induction. (A)** ”High-score” patients had significantly lower delta-band power starting around 3 to 5 min after LOR. **(B)** ”High-score” patients did not have significantly higher theta-band power apart from a short episode around 10 min after LOR. **(C)** ”High-score” patients had significantly higher alpha-band power throughout induction. **(D)** ”High-score” patients had significantly higher beta-band power starting around the time of LOR. **(E)** ”High-score” patients had significantly lower gamma-band power starting around 5 min after LOR. **(F)** AUC values as the effect size are symmetrical around 0.50, therefore both AUC < 0.30 or > 0.70 are considered acceptable.

When correcting for the differences in total power, i.e., by using the normalized power, the differences between the groups were less prominent and revealed the effect of overall power. In relative terms, power of the ”high-score” group was lower in the delta and gamma-band starting around 3 to 5 min after LOR. Beta and alpha power, on the other hand, were higher starting around the time of LOR for beta and, to a certain degree, already before in the alpha-band. Theta power did not show any relevant differences apart from a short episode around 10 min after LOR (Fig. 4).

#### 3.3.3 Separation performance at LOR

As indicated in (Fig. 3) some parameters can already separate the two groups before LOR. Similar to the time point at composite score calculation, AUC values below 0.3 can be observed for all bands (including total power) except gamma. Also, SEF before LOR was not as good at separating the groups as SEF at the timepoint of composite score computation (after LOR). AUC values for both time points can be found in Table 2.

**Table 2:** AUC values before loss of responsiveness (LOR) and at the time when the composite score was calculated, including 95 % confidence interval for the separation of ”high” and ”low” score patients. AUC values as the effect size are symmetrical around 0.50, therefore both AUC < 0.30 or > 0.70 are considered acceptable.

	**AUC before LOR**	**AUC at Composite Score**
**Alpha**	0.23 [0.117, 0.343]	0.03 [0.001, 0.076]
**Total**	0.24 [0.136, 0.367]	0.03 [0.000, 0.077]
**Propo**	0.27 [0.142, 0.398]	0.27 [0.146, 0.397]
**SEF**	0.60 [0.442, 0.756]	0.27 [0.152, 0.394]
**Delta**	0.29 [0.169, 0.419]	0.09 [0.026, 0.175]
**Theta**	0.24 [0.130, 0.365]	0.08 [0.023, 0.146]
**Beta**	0.27 [0.148, 0.410]	0.04 [0.010, 0.092]
**Gamma**	0.48 [0.331, 0.624]	0.20 [0.107, 0.309]

#### 3.3.4 Beyond dichotomization

**Fig. 5:**
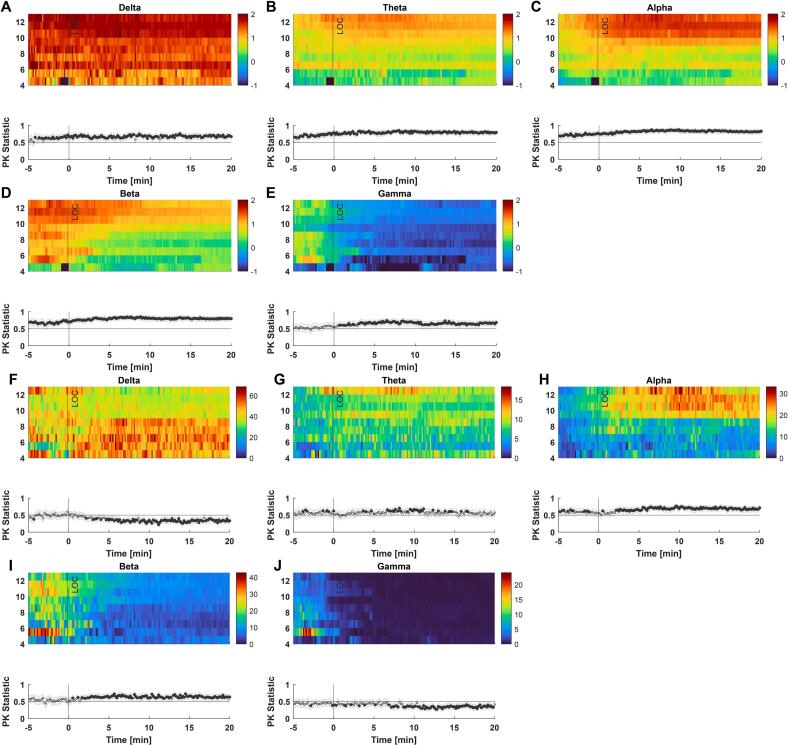
**Differences in absolute (A-E) and relative (F-J) EEG band power across different levels of composite score values. (A)** Patients with higher composite scores had significantly higher delta-band power throughout induction. **(B)** Patients with higher composite scores had significantly higher theta-band power throughout induction. **(C)** Patients with higher composite scores had significantly higher alpha-band power throughout induction. **(D)** Patients with higher composite scores had significantly higher beta-band power throughout induction. **(E)** Patients with higher composite scores had significantly higher gamma-band power starting around the time of LOR. **(F)** Patients with higher composite scores had significantly lower delta-band power starting around 3 to 5 min after LOR. **(G)** Patients with higher composite scores did not have significantly higher theta-band power apart from a short episode around 8 to 12 min after LOR. **(H)** Patients with higher composite scores had significantly higher alpha-band power after LOR and partly at induction. **(I)** Patients with higher composite scores had significantly higher beta-band power starting around the time of LOR. **(J)** Patients with higher composite scores had significantly lower gamma-band power starting around 7 min after LOR. AUC values as the effect size are symmetrical around 0.50, therefore both AUC < 0.30 or > 0.70 are considered acceptable.

Higher scores were generally associated with higher absolute power throughout the duration of the recording. Only gamma power before LOR was similar across the different levels of the composite score (Fig. 5 **A-E**). Relative band power, on the other hand, was associated with higher power in the alpha and beta-band, while delta and gamma were lower when patients had higher composite scores (Fig. 5 **F-J**). This was also true when we break traditional band power limitations and look at density spectral arrays (**Fig. S1**).

## 4 Discussion

Composite scores like the BPTIVA were presented as a valuable tool for risk stratification regarding burst suppression and/ or postoperative cognitive outcomes (Touchard et al., 2019). The idea of such an index is appealing, but its application has several limitations regarding applicability and reproducibility. The authors define thresholds for SEF, total power, and alpha-band power. Technical features like sample rate, filters used, or the distance between recording and reference electrode can influence EEG power estimation and consequently these features. So, the presented thresholds are only valid for the specific setting the authors chose. We tried to reproduce the composite index and, with some modifications, could show very similar results. Even though we found similar behaviors, we feel the need to highlight the importance of carefully selecting the sub-components that make up the overall score. The inclusion of more than one spectral based parameter, such as total power and absolute alpha power, results in issues of multicollinearity. Similarly, most spectral-based parameters are driven by the patients age, as expected from previous studies (Purdon et al., 2015a, Purdon et al., 2015b, Schultz et al., 2004), and not necessarily ”frailty”. The same holds true for the propofol requirement, i.e. older patients require less (Schnider et al., 1998). Consequently, age seems to be the main determinant of the composite score, the BPTIVA score, and the cognitive reserve. Therefore, the BPTIVA score should not be interpreted as a frailty marker until age-adjusted validation is performed. Similarly, future scores need to be corrected for age if they aim towards identifying a frail brain. In contrast to the original study (Touchard et al., 2019), none of our included patients experienced induction burst suppression, probably as a result of the slowly titrated induction. Hence, we could not reproduce the relationship between the composite score and burst suppression. This observation of reproducing the score’s behavior but without the occurrence of the ”fragility” marker, i.e., burst suppression, questions the score’s ability to identify these ”fragile” patients.

Our results point towards the direction of the score being biased by other factors such as age. However, by using the composite index to dichotomize patients into ”low-score” and ”high-score” patients, we could show that differences in EEG features were already prominent before loss of responsiveness and during anesthesia induction, i.e., before the intraoperative period that was used for creating the score. The slow and prolonged induction also allowed a more nuanced analysis of anesthetic-induced EEG dynamics. Hence, we were able to describe the development of significant differences in the (relative) band-power between the ”high-scorers” and ”low-scorers”. These results indicate that the development of intraoperative EEG features may already be recognized during the (slow) induction phase. An early identification of such differences may help to optimize and adjust anesthesia navigation ”before the horse has bolted”, i.e., before the patient enters excessively deep anesthesia levels. Findings from preoperative awake baseline recordings already describe EEG differences in patients who did or did not develop signs of delirium in the postanesthesia care unit (Schüßler et al., 2023, Koch et al., 2021). In this context, the non-appearance of burst suppression in our study shows that the titration setting may be key to defining a patient as vulnerable or not. Rapid and high doses that produce a bottom-up type LOR are related to burst suppression occurrence both by cortical overdose and by the mechanism of discouraging thalamocortical circuits that sustain multistability (Sepulveda et al., 2020). The presence of burst suppression in this scenario may be more associated with the degree of overdose and not necessarily with brain vulnerability (Hesse et al., 2019, Lutz et al., 2022). Touchard’s original study uses Schnider’s PKPD model for propofol to define its effect site concentration at LOR after loading at 5 µg/ml in TCI. The Schnider model has shown a significant overprediction (Glen and Servin, 2009, Sepulveda and Mora, 2012), which becomes obvious by the large difference in effect site concentration we observed in our study following a slow induction protocol. The choice of PKPD model and induction speed may influence the effect site concentration at LOR and the development of burst suppression during the induction period. Other changes that were visible both in the dichotomized and non-dichotomized analysis, was the overall increase in absolute power for higher scoring patients as well as in relative terms the increase in alpha power. The clinical significance of intraoperative alpha power is an evolving area of investigation, and a causal link between alpha power and outcome has not been shown. However, a strong alpha and delta pattern seems to be a key EEG signature of propofol in healthy patients (Purdon et al., 2015a, Purdon et al., 2015b). Moreover, reduced intraoperative alpha power has been linked to frailty, cognitive impairment, and an increased risk of postoperative delirium (Giattino et al., 2017, Gutierrez et al., 2019). A strong alpha oscillatory activity also seems to carry some information about the nociceptive state of a patient, as alpha EEG features changed when analgesia may have been insufficient (Hight et al., 2019, Hagihira et al., 2004). Although we could not track postoperative outcome in this analysis, early divergence in alpha and/or beta power could potentially flag brains that will later show greater anesthetic sensitivity. Yet this hypothesis warrants prospective testing.

## 5 Limitations

Of course, there are numerous limitations to be considered. As already mentioned, most composite scores are designed on a defined set of data, and the chosen, maybe arbitrary, settings only apply to the respective data set. So, we are, to some degree, comparing apples to oranges, especially since the endpoint of one composite score, burst suppression, did not occur in our data. As we could present similar trends, we would like to question the usefulness of these scores in detecting a frail brain. They probably only detect an ”older” brain as the correlation of the composite score and its sub-components showed. But further investigation is necessary to further understand the age-dependency and the potential influence of other factors. Therefore, we do not need to wait until after LOR, as our induction trends show. Further, we did not evaluate postoperative cognitive outcomes and cannot make any assumptions about the correlation between observed EEG differences during induction and a postoperative neurocognitive disorder. This is a major limitation and a significant knowledge gap. Consequently, as we did not measure outcomes or anesthetic dose adjustments, clinical utility of our composite score remains hypothetical. Further, the original score was designed as a “fragility” marker, which was associated with the occurrence of burst suppression in the EEG. With our data, we can only replicate some of the composite scores’ behavior, e.g. variations of the score itself between patients or its association with age. Since none of the patients presented with burst suppression episodes, the fragility endpoint was missing and could therefore not be replicated. Additionally, we needed to change the scoring system in order to be able to at least partially replicate the BPTIVA approach. Opening the upper boundary of the SEF95 criterion may bias our results towards time points where the EEG is less dominated by lower frequencies. Consequently, scores may be calculated at a different level of ”depths/ cortical depression” as opposed to the reference by Touchard and colleagues (Touchard et al., 2019), ultimately reducing the comparability of the results. We chose an observational window from 5 min before to 20 min after LOR. Our results are consequently only valid for early intraoperative periods.

## 6 Conclusion

With our analyses, we could show two things. First, the use of composite scores, including different age-dependent features, does not seem to be a marker of brain frailty as identified by burst suppression occurrence during (rapid) induction; rather, it is only a marker of age. Second, the group differences observed after induction, as included in the scores, are already identifiable through a slow induction protocol. Hence, the assessment could have already been performed at an earlier time. We also want to argue that evaluation of frailty or other unwanted intraoperative outcomes (e.g., low alpha power) should be evaluated in controlled scenarios. Rapid induction using boluses faces a scenario of so-called kinetics of the border with high instability of drug mixtures. Progressive titration of anesthesia may not only be helpful for such research purposes, but also for clinical practice. At those slower timescales, it is often easier to observe and evaluate patient responsiveness, while at the same time allowing the EEG to catch up, such that it can be used for confirmation purposes. Nevertheless, future work should test whether the same age-bias appears under standard rapid-induction protocols.

## Author contributions

All authors have approved the final version of the manuscript. **JO:** This author helped to analyze the data and to write the manuscript. **AM:** This author helped to analyze the data. **DO:** This author helped to discuss the results, and write the manuscript. **GS:** This author helped to discuss the results, and write the manuscript. **PS:** This author collected the data, helped to discuss the results, and write the manuscript. **MK:** This author helped to analyze the data, discuss the results, and write the manuscript.

## Declaration of competing interest

The authors declare the following financial interests/personal relationships which may be considered as potential competing interests: The authors declare that this research did not receive any other specific grant from funding agencies in the public, commercial, or not-for-profit sectors. Matthias Kreuzer is named as an inventor for a patent dealing with spectral EEG features and age (U.S. Provisional Patent Application No. 62/914,183). Gerhard Schneider and Matthias Kreuzer are named as inventors for a patent filed on a novel method for intraoperative EEG monitoring (U.S. Patent Application Serial No. 62/960,947). Gerhard Schneider, Matthias Kreuzer are also named as inventors for a patent dealing with the EEG features during anaesthesia emergence (U.S. Provisional Patent Application No. 63/459,294). Matthias Kreuzer received funding from Masimo Corporation, Narcotrend-Gruppe, Medtronic GmbH and Fresenius Kabi Deutschland GmbH for conducting EEG-based training for anaesthesiologists and received honoraria for speaking engagements related to the EEG.
