# Supplementary material for: Age-dependent changes in the power spectrum conflate composite scores to assess brain frailty
Source: Clin Neurophysiol Pract. 2025 Jun 19;10:209–17. doi: 10.1016/j.cnp.2025.06.002 (PMC12246862; doi:10.1016/j.cnp.2025.06.002)
Supplement: Supplementary Data 1 [file mmc1.docx]

| **Score** | **1** | **2** | **3** | **4** | **5** |
| --- | --- | --- | --- | --- | --- |
| **Propofol Ce LOR** | < 2.5 | 2.5 – 3.5 | >= 3.5 | - | - |
| **Total Power (*µ*V^2^)** | < 800 | 800 – 1500 | 1500 – 3000 | > 3000 | - |
| **Alpha Power (*µ*V^2^)** | < 100 | 100 – 200 | 200 – 300 | 300 – 400 | > 400 |

**Table S1**: Subcomponents of the composite score and their individual contributions
