# Supplementary figures and images for: Age-dependent changes in the power spectrum conflate composite scores to assess brain frailty
Source: Clin Neurophysiol Pract. 2025 Jun 19;10:209–17. doi: 10.1016/j.cnp.2025.06.002 (PMC12246862; doi:10.1016/j.cnp.2025.06.002)

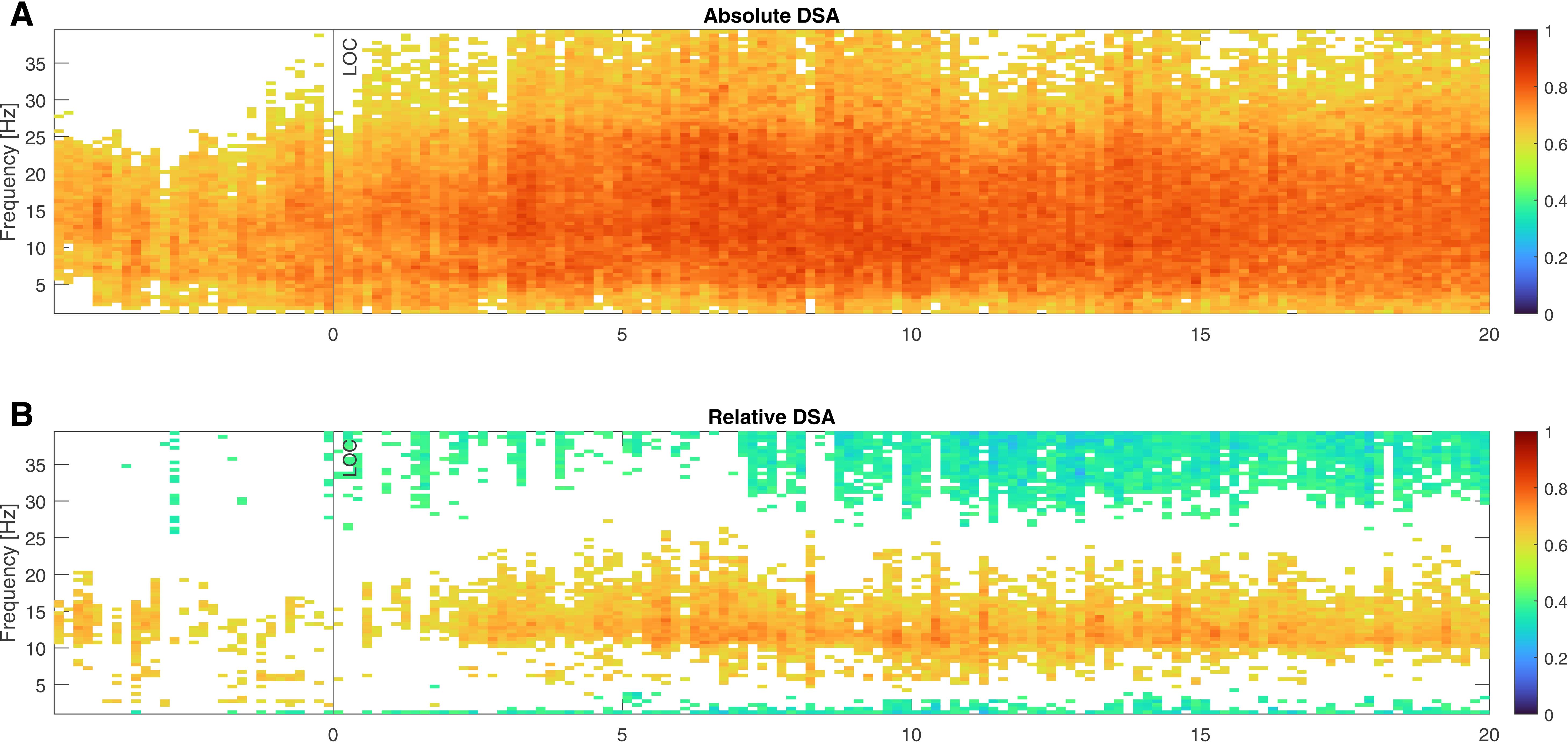

Supplement: Supplementary Fig. 1 [file mmc2.jpg]
